# Progress in Medicine

**Published:** 1896-08-29

**Authors:** 


### Progress in Medicine.

### INFECTIOUS DISEASES.

**Scarlet Fever.**—Marmorek[1] treated ninety-six children with antistreptococcal serum. All were suffering from scarlet fever and showed the presence of the streptococcus; seventeen of the number had Lœffler's bacillus also. Ten centigrammes daily were usually injected into each patient, who thus received from ten to ninety centigrammes in all. Though the eruption and pyrexia of pure scarlatina were unaffected, the complications, swollen glands, and faucial troubles quickly yielded. However, there appear to be different varieties of the streptococcus, some of which are unaffected by the serum. Thus Méry[2] found one immune to it in a scarlatinal patient, and others have been isolated in animals. Baginsky[3] remarked in forty-eight cases treated with the serum that the course and mortality were favourably affected, though in some instances it appeared useless. The results seem to call for a more extended trial of the remedy, but point strongly to the conclusion that streptococcus is not the cause of the disease pure and simple, but rather a common complication. Thus, Lemoine[4] tested the blood of thirty-three patients in normal scarlatina without finding the coccus, but it was present in two hæmorrhagic cases and in pleuritic fluid, as well as in the urine of every one of twenty-two cases of albuminuria. The renal complications thus appear to be entirely due to it, though Widal believes they are produced by the scarlatinal germ itself. Among other authors who have sought for the true microbe of scarlatina quite recently, Crajkowsky[5] cultivated certain diplococci found in the blood of scarlatinal patients, and stained them by Chencinski's formula. They form minute points on solid media, do not stain by Grain's method, and are of slow growth; destroy mice when inoculated but have no effect on rabbits.

At the British Institute of Preventative Medicine two horses have been rendered immune against the streptococcus, and two against that organism and Lœffler's bacillus. The virulence was increased by passing the cultivation through rabbits, and was originally derived from an erysipelas patient. As a medium of growth ascitic fluid mixed with peptonised bouillon was found to be satisfactory. Bulloch[6] remarks that for the production of highly immunised serum the horses require twelve months' treatment, and that the true clinical value of the remedy will be rendered uncertain for some time from the difficulty of diagnosing pure streptococcal diseases. Thus staphylococcal infections have been treated with no effect by the serum. The same writer gives a *resumé* of the various diseases in which the streptococcus plays a part.[7] He considers that the identity of S. pyogenes with S. erysipelatosus and puerperalis is completely proved, but that the organism varies astonishingly in virulence, and according to the site of inoculation, and with the presence or absence of associated microbes. Its origin may be from without, or in some cases from harmless forms existing in the body, but intensified by the reaction of other germs, such as the typhoid one, or by diminished resistance of the body. Besides erysipelas we find streptococcal phlegmon of the skin, and varieties of pyæmia, septicæmia, and puerperal sepsis. The latter may be caused also by gonococci, bacillus coli, and other organisms, though not by the putrefactive ones alone. The streptococcus is the most frequent cause, and may be external or internal in its origin.

Many cases of bronchitis, pneumonia, both lobar and lobular, and empyæma are caused by it, and it often intensifies an influenza. The hectic fever of phthisis is probably a symptom of its presence, to which a large part of the mortality is due. So, too, certain forms of otitis, meningitis, enteritis, liver disease, nephritis, and Landry's paralysis are due to it, while typhoid, variola, and scarlatina are intensified by its action. Hence the enormous importance of the organism and the need of some means of coping with it.

The mortality of scarlatina in the Children's Hospital, Rue de Sevres, Paris,[8] was only 5·85 per cent. last year. In only two cases was Marmorek's serum used, and then with little effect. Albuminuria occurred in 20 per cent., but anasarca only in 6. Rendu and others[9] regard cases of recurrence or relapse during convalescence as generally due to streptococcal infection, as they are usually associated with complications such as otitis. T. O. Symes[10] describes two cases of scarlatina during pregnancy. Parturition took place while the infection was present, antiseptic precautions were employed with success, and no septicœmia followed. There is, indeed, no relation between pure scarlatina and puerperal sepsis beyond the danger of vaginal inoculation from the streptococci generally present. Duckworth[11] considers that the arthritis of scarlatina may either be rheumatic or septic, the latter being analogous to gonorrheal, and the former being of the ordinary type. Epilepsy occurs as a sequel more often than after any other infectious disease.

**Cholera.**—The great outbreak in Egypt was preceded by isolated cases as far back as September and October of last year, which Rogers Pasha[12] insists were derived from the Mecca pilgrims, and not from the limited outbreak in Asia Minor. It appeared in Alexandria in March, and soon after in Cairo, but only 115 cases were found in all Egypt up to the middle of May[13]. The way in which sanitary reforms have been allowed to lag behind other improvements in the country becomes a serious matter at this time, but the hindrances to official action are undoubtedly very great. As the summer has gone on the disease has spread, and 5,193 cases, with 4,776 deaths, were recorded up to the middle of June[14], and soon after July over 14,000 deaths were registered. In Cairo and Alexandria the disease seemed to be brought to an end by the efforts of the officials[15], but the expeditionary army was attacked in the Soudan, and several British officers died, as well as more troops than had been killed in battle. Powell[16] gives the results of some of Haffkine's inoculations in Assam. In a population of 3,150 uninoculated, there were twenty-eight and a-half cases of cholera per thousand, and a death-rate of 17·4; while among a nearly equal number of inoculated persons there were 3·2 cases per thousand, and a death-rate of 1·4. In other localities the comparative death-rates were 36·5 and 5·3; but where smaller doses with little or no febrile reaction were used, as in the East Lancashire Regiment, the rates were 17·3 and 9·5. Thus the writer concludes that the strongest doses do not give complete immunity, though a high one, while small ones give little protection.

The late Sir George Johnson,[17] shortly before his death, published his "History of the Cholera Controversy," and addressed a final letter to the *Lancet*, attacking the treatment by opium. In it he shows that recent experiments bear out his view that to lock up the contaminated secretions in the body is the surest way to destroy all chance of recovery. He insists on the value of an initial purge by castor oil, and on the danger of opium increasing collapse by lessening the pulmonary oxidation. Gruber[18] conferred on guinea pigs immunity by the use of sterilised cultures of the vibrio, and found they acquired the power of destroying the microbes, but were not protected against poisoning by the toxin. This, of course, is the opposite of what is found in diphtheria. The same result is also seen in typhoid immunity. He noticed, too, that the first effect of the serum is to render the microbes motionless, and to cause them to stick together in solid masses. This reaction, which is specific for each disease, furnishes a useful diagnostic test, so that serum immunised for cholera or one prepared for typhoid will show the presence of the germ against which it is a protective by this "agglutinising" power.[19] The virulence of the cholera bacillus, according to Blackstein, may be *nil* until it has been grown in a medium containing phosphate of iron or bile. Hence the value to it of a sojourn in the soil, and the small virulence seen when passed direct from one patient to another.

However, a controversy has arisen whether toxin obtained from cultures is the same as that in the bodies of the microbes, and whether antitoxins prepared from each may not have very different properties. Thus, Metchnikoff and Roux[20] claim to have prepared from cultures a toxin resistant to heat, and by it again a protective serum which neutralises the poison, though apparently not destructive to the microbes. Pfeiffer's serum prepared by injection of the bodies of bacilli, on the other hand, destroys fresh bacilli, though it is powerless against the poison. Thus, the difference in the action of cholera, typhoid, and diphtheritic serums depends on the mode of preparation. Two kinds can be obtained, the one destroys the invading microbes and the other neutralises their products. Stutzer[21] reaches the conclusion that cholera vibrios are rapidly destroyed in water containing fæces and urine, but live much longer where these are absent or present in very small proportions. Most observers will regard this, however, as needing much corroboration to be accepted.

[1] Internat Medic. Mag., April. [2] Med. Week., April 24. [3] B.M.J., May 16; Lanc., May 9. [4] Med. Week., March 27. [5] Med. Record, April 18. [6] Lancet, May 2. [7] Lancet, April 11. [8] Lancet, May 16. [9] Med. Week., June 12. [10] Med. Press, June 24. [11] B.M.J., April 4. [12] B.M.J., April 18. [13] Lancet, May 30. [14] Lancet, June 27. [15] B.M.J., July 11. [16] Lancet, July 18. [17] Lancet, June 13. [18] Med. Week., March 6. [19] Med. Week, April 24. [20] B.M.J., June 20. [21] B.M.J., July 25.

